# T Lymphocytes and Testicular Immunity: A New Insight into Immune Regulation in Testes

**DOI:** 10.3390/ijms22010057

**Published:** 2020-12-23

**Authors:** Jialei Gong, Qunxiong Zeng, Di Yu, Yong-Gang Duan

**Affiliations:** 1 Shenzhen Key Laboratory of Fertility Regulation, Center of Assisted Reproduction and Embryology, The University of Hong Kong-Shenzhen Hospital, Shenzhen 518053, China; gongjl@hku-szh.org (J.G.); zengqunxiong@icloud.com (Q.Z.); 2 The University of Queensland Diamantina Institute, Faculty of Medicine, The University of Queensland, Woolloongabba, QLD 4102, Australia; di.yu@uq.edu.au

**Keywords:** spermatogenesis, regulatory T cells, helper T cells, cytotoxic T cells, γδ T cells, natural killer T cells, Leydig cells, Sertoli cells

## Abstract

The immune privilege of the testes is necessary to prevent immune attacks to gamete-specific antigens and paternal major histocompatibility complex (MHC) antigens, allowing for normal spermatogenesis. However, infection and inflammation of the male genital tract can break the immune tolerance and represent a significant cause of male infertility. Different T cell subsets have been identified in mammalian testes, which may be involved in the maintenance of immune tolerance and pathogenic immune responses in testicular infection and inflammation. We reviewed the evidence in the published literature on different T subtypes (regulatory T cells, helper T cells, cytotoxic T cells, γδ T cells, and natural killer T cells) in human and animal testes that support their regulatory roles in infertility and the orchitis pathology. While many in vitro studies have indicated the regulation potential of functional T cell subsets and their possible interaction with Sertoli cells, Leydig cells, and spermatogenesis, both under physiological and pathological processes, there have been no in situ studies to date. Nevertheless, the normal distribution and function of T cell subsets are essential for the immune privilege of the testes and intact spermatogenesis, and T cell-mediated immune response drives testicular inflammation. The distinct function of different T cell subsets in testicular homeostasis and the orchitis pathology suggests a considerable potential of targeting specific T cell subsets for therapies targeting chronic orchitis and immune infertility.

## 1. Introduction

Mammalian spermatogenesis starts from undifferentiated spermatogonia, which then undergo reduction division, finally forming spermatozoa. Along with their enormous morphological transformation and genetic material shift from diploid to haploid, gene expression profiles and cell surface proteins also change significantly [1]. This progress, however, creates great challenges for the immune system in the testes, as these differentiated spermatozoa are not generated until puberty, long after the establishment and maturation of the immune system, including the tolerance to self-antigens. As a result, the exclusively expressed proteins on the surface of the sperm membrane can be antigenic and immunogenic for the body. To avoid autoimmune attack on sperm and protect the spermatogenesis, the testes develop a set of mechanisms to create a haven for these cells, thus preserving the reproductive capacity of men. The blood–testis barrier (BTB) mainly consists of Sertoli cells (SCs), which form a physical shield for sperm that circumvents the immune cells and provide a protective biochemical environment [2]. While this physical barrier efficiently prevents the immune–sperm cell contact, other immune mechanisms are also required to inhibit the potential autoimmune response, especially at some joints where the dam is frail or missing, such as the tubuli recti and rete testis. These mechanisms include a diminished reactivity of resident macrophages to inflammation, inhibited proinflammatory cytokines, tolerogenic cytokines induced by androgens, anti-inflammatory cytokines expressed by regulatory immune cells and somatic cells, antigen-specific immune suppression by dendritic cells, and quiescent mast cells [3]. All of these sophisticated designs, together with BTB, form an immunologically privileged site in the testes that guards the sperm.

In terms of physiological conditions, the immune privilege provides a unique environment for suppressing the immune response and sustaining normal spermatogenesis [4]. However, the testicular environment does not entirely preclude inflammation or immune responses, which might have a destructive impact on spermatogenesis when it is challenged by virus or bacteria [5,6]. Previous studies have shown that infection and inflammation of the male genital tract is a frequent cause or cofactor of male infertility, accounting for about 25% of male patients undergoing testicular biopsy and approximately 6–15% of all cases of fertility disturbances [7,8,9]. Infection and acute or chronic inflammation are thought to impair the function of the testes and epididymis through the inhibition of steroidogenesis, induction of germ cell (GC) apoptosis, and indirect effects on the activity of the epithelial cells, thus causing infertility [10].

T lymphocytes are the central regulator of immune response and play a key role in inflammation. The involvement of T cells has been widely detected in chronic inflammation, especially in some autoimmune diseases such as rheumatoid, where CD4^+^ T cells play an orchestral role. T cells function directly in a contact-dependent manner or indirectly by secreting soluble mediators. Several different subsets of T cells have been accumulatively detected in the testes, including regulatory T (Treg) cells, helper T (Th) cells, cytotoxic T (Tc) cells, natural killer (NK) T cells, and γδ T cells (Table 1). While these cells only account for the minority, for example, 10–20% of total immune cells in adult rat testes under physiological conditions [11], they are essential for the maintenance of immune homeostasis and are involved in the pathogenesis of male infertility [12].

The infiltration level of T cells is assumed to be concurrent pathologies of the testes, such as seminiferous tubular damage, thickening of the lamina propria, and complete tubular fibrosis [24]. The systematic immunohistochemical analysis of testis biopsies from infertile patients with azoospermia indicated that immune cells were mainly infiltrated by CD3^+^ T cells, instead of CD20^+^ B cells and CD11c+ dendritic cells, regardless of the degree of inflammation [25]. In patients with a low testicular volume, a positive linear relationship was observed between CD45 lymphocytes infiltration and sperm parameters, such as the percentage of immature germ elements, phosphatidylserine externalization, early apoptosis, and abnormal morphology [26]. In a systematic re-examination of tissue samples obtained from chronic orchitis with infertility, the infiltration of T lymphocytes was recorded in as many as 56.3% of cases [27]. Moreover, several experimental models show that the repeated inoculation of tissue antigen (Ag), especially the Ag of immune-privileged organs, such as the testes, can induce organ-specific autoimmune diseases in mice, such as experimental autoimmune orchitis (EAO), a model of chronic inflammation used for investigating the pathogenic mechanism involved in testicular damage. Terayama et al. found that subcutaneous immunization with testicular germ cells (TGC) can induce EAO without adjuvants, and TGC-elicited EAO exclusively responds to haploid germ cells without depleting diploid germ cells or affecting the epididymis or vas deferens [28,29]. γδ T cells, a small group of T cells, can recognize self-peptides and, in many respects, are implicated in autoimmune diseases and also involved in testicular inflammation [30]. These findings indicate the resident autoreactive T cells in normal testes and/or tissue-specific recruited T lymphocytes as a crucial component of the inflammation cascade in the testes.

By addressing the relationship between male infertility and immune dysfunction, we can better understand how the immune system operates and functions in spermatogenesis. The mechanism associated with this relationship will also aid in the search for effective targets for drug discovery. In view of the core status of T cells in immunoregulation, in this review, we aim to summarize the studies and advance the research on testicular T cells in terms of the physiological content and the pathogenesis content of orchitis, considering T cells in terms of the therapeutic strategy for orchitis and infertility.

## 2. T Lymphocytes in the Testes

### 2.1. Regulatory T (Treg) Cells

Treg cells are a group of regulatory T cells that are essential for immune tolerance in peripheral blood and some solid organs. They are devoted to preventing excessive immune response and autoimmunity by suppressing other reactive T cells. In recent decades, preclinical studies and laboratory research have been conducted to investigate the ability and characteristics of Treg cells, as a consequence of which our understanding of their differentiation and distribution has increased considerably. Generally, while mouse Tregs can be sorted by the CD25 and FoxP3 markers, there are no sufficiently specific markers for human Tregs, as some effector T cells may upregulate these markers after stimulation as well [31]. However, the upregulated Foxp3 level in effector T cells is transient and relatively lower than the significantly high, stable expression of Fox3p in Tregs [32]. In contrast, although CD25 was found to be highly expressed in the majority of Tregs, CD25^−^Foxp3^+^ Tregs can be detected in secondary lymphoid organs and other tissues. Both of these two subtypes of Tregs have a similar transcriptional profile and suppressive function [33]. Recently, in gene-engineered mice, which showed a real-time Foxp3 expression, the Foxp3 level was shown to present a dynamic pattern during inflammation, and these dynamics determine the effector Treg program [34]. In general, Foxp3 can act as an effective suppressive marker in most Tregs, and regardless of the heterogeneity of Tregs, their various mechanism of suppressive function can be classified into four basic ‘modes of action’: secreting inhibitory cytokines, including IL-10, IL-35, and TGF-β, cytolyzing activated T cells, disrupting the metabolic process, and modulating the maturation and function of dendritic cells [35]. While numerous studies have been conducted to investigate the function and differentiation of Tregs, limited data are available on the function of Tregs in the testes and their association with fertility. An in vitro test, mice model, and clinical examination will help us to learn their vital role in these immune privilege sites.

Treg cells have been found in the testes of mice [14,36], rats [12], and humans [13]. While no clear evidence shows the origin of these Tregs, they may mainly reside in draining lymph nodes of the testes (TLN), where tissue-specific autoantigens continuously interact with them and maintain their suppressive function [37]. This hypothesis was consolidated by an experiment on autoimmune orchitis, as CD4^+^CD24^+^Foxp3^+^ Tregs were shown to be increased in TLN, rather than the lymph nodes from the immunization site (ILN) [38]. Defects in testicular Tregs lead to abnormal immune response and aggravate autoimmune orchitis. In particular, patients with autoimmune regulator gene (AIRE) mutation, which causes autoimmune polyendocrine syndrome-1, are prone to chronic testicular inflammation due to deficient Tregs [39]. In vasectomized mice, CD4^+^CD25^+^ Tregs mediated immune tolerance to meiotic germ cell antigens (MGCA), which egressed from normal tubules, while depletion resulted in MGCA-specific autoimmune response and bilateral orchitis [36,40]. Therefore, Tregs are critical for preventing organ-specific autoimmunity and maintaining the immune privilege of the testes.

**Figure 1:**
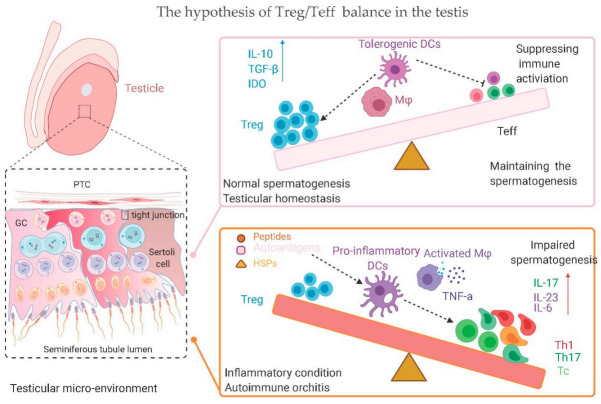
Balance between Treg cells and Teff cells in the testes. Under normal conditions, Tregs could effectively control Teff cells through several immunosuppressive mechanisms, and a balance between them could instrumentally sustain and foster normal spermatogenesis and testicular homeostasis. However, Treg cells are outnumbered by vigorously expanding Teff cells under pathological states, such as testicular chronic inflammation and infection, and the balance between Treg and Teff cells is disturbed, resulting in impaired spermatogenesis, autoimmune orchitis, and/or azoospermia. DC, dendritic cell; HSP, heat shock protein; IL, interleukin; IDO, indoleamine 2,3-dioxygenase; Mφ, macrophage; Treg, regulatory T cell; Teff, effective T cell; TGF-β, transforming growth factor β; TNF-α, tumor necrosis factor α; Upward arrow: detectable increase of cytokine level; Dotted line: proposed communication among cells.

In testes undergoing autoimmune orchitis, either due to pathological inflammation or EAO, the quantification of immune infiltrating cells shows an alternation in Treg subsets. Interestingly, while our data show decreased Foxp3^+^ Tregs in human azoospermic testes with chronic inflammation (ATCI) and increased levels of IL-17, Jacobo et al. (2009) reported a fluctuation of CD25^+^Foxp3^+^ Tregs in EAO, showing an increase in both CD8^+^CD25^+^Foxp3^+^ and CD4^+^CD25^+^Foxp3^+^ at the EAO onset, following a decrease in the latter through the chronic phase [12,13]. While differences between rats and humans could explain this discrepancy, it is most likely due to the different panel of markers used to detect Tregs, as we only stained Tregs with Foxp3^+^, which includes the two aforementioned subsets of Tregs. It is reasonable to assume that the decreased number of CD4^+^ Tregs in the chronic phase of orchitis outnumbers the increased number of CD8^+^ Tregs, thus representing an overall decrease. The Th17-promoted milieu and shift among Treg subsets may undermine the ability of Tregs to suppress inflammation in the testes, because heterogeneous Tregs exhibit very different regulatory efficiencies. Jacobo et al. demonstrate the functionally effective state of CD4^+^ Tregs [38]; however, Treg cells accumulated in inflamed testes are unable to downregulate inflammation [12,41]. CD8^+^ Tregs may possess a relatively lower suppressive character, and the imbalance in Treg subsets leads to deficient immunoregulation, which can trigger autoimmune disease [42]. In addition to the subset shift of Tregs, there are some other underlying mechanisms that can be attributed to Treg deficiency: (a) a skewed population of Tregs and effector T cells; (b) the Treg-tolerance of pathogenic T cells, such as Th17; (c) the inhibitory effects of local proinflammatory cytokines on Tregs, such as TNF-α and IL-6 (Figure 1) [43,44]. An investigation that addresses these issues may contribute to the Treg-based therapy of impaired spermatogenesis and autoimmune orchitis. For example, the removal of proinflammatory cytokines or the addition of inhibitory cytokines, before or after the administration of Tregs, may improve treatment outcomes.

### 2.2. Helper T (Th) Cells

T helper cells are a large subgroup of T cells originating from naïve T cells (generally known as CD4^+^ T cells). While Tregs are defined by transcription factor Fop3, and T follicular helper (Tfh) cells are defined by the combination of a surface marker and follicular localization, other Th cells are mainly defined by cytokines secretion. These subsets include Th1, Th2, Th17, and some additional unconventional subsets, such as Th3, Th9, Th22, and T regulatory type1, which, together with Tfh cells, have not been identified in the testes. Th1 cells are mainly involved in antiviral and antibacterial immunities by producing the cytokine interferon-γ, interleukin (IL)-2, and tumor necrosis factor (TNF)-α. Th2 cells are supposed to play an immunologically important role in the fight against extracellular pathogens, like some parasites, and this subset of cells is designated by their signature cytokines, IL-4, IL5, and IL-13. Th17 cells are essential for antifungal function and defense against infection of bacteria, which are accomplished by secreting inflammation cytokines, IL-7A, IL-17F, and IL-22. There are several notable differences in the differentiation of Th17 cells between humans and mice, which might have clinical implications. For instance, while IL-6 is essential in mice, it only plays a supportive role in humans; the opposite is the case for IL-23 [45]. Another difference between humans and mice in terms of Th17 is the impact of IL-2. While IL-2 impinges IL17 production in mice, IL-2 promotes the generation of human Th17 cells in vitro, and the use of IL-2 to treat HIV-infected patients did not affect the population of Th17 in blood [45]. In general, these helper T cells usually exert an immune response indirectly, such as facilitating immunoglobulin switching in B cells, activating and recruiting CD8^+^ T cells, and magnifying the bactericidal activity of phagocytes [46]. An adequate number and activity of Th cells are necessary for maintaining homeostasis in the testes. While immune tolerance is essential for spermatogenesis, as an immune privilege site, it also puts the testes at a high risk of infection and tumorigenesis. On the other hand, an excessive immune activity of Th cells might evoke an immoderate immune response and inflammation, which would accelerate the progress of chronic orchitis. Therefore, a clear profile of Th cells in the testes may help us to understand the dynamic equilibrium or unbalance during homeostasis or inflammation, which would contribute to an appropriately clinical scheme of drug intervention.

Early studies showed that Th1 cells secrete Interferon-γ (IFN-γ) in mouse [16], rat [17], and human testes [15], while Th2 cells might not be present in mouse and human testes, since their cytokine-producing IL-4 could not be detected [13]. However, recently, the mRNA of IL-4 was detected in rat testes undergoing experimental autoimmune encephalomyelitis (EAE) [47]. Nevertheless, these results clearly suggest that Th1, but not Th2, cells are involved in the development and maintenance of autoimmune orchitis. Th17 cells were later detected in the testes of the EAO rat as well. Together with Th1, Th17 cells predominate in the testes during EAO onset; however, in the chronic phase, CD8^+^ T cell-producing IFN-γ and IL-17 represented the major subset, while maximizing the IL-17 and IL-23 level [19]. The population shift of Th1 and Th17 cells suggests a dynamic composition of Th cell subsets during orchitis progress. For Th17 cells, the mRNA quantification of IL-23p19 in the TLN of control and experimental rats shows stable transcription during the initiation of EAO, which indicates a different pattern of Th17 activation in the lymphoid organ inside the testes [48]. Moreover, the data from the mouse model comprehensively support the dynamic pattern of Th cells: (1) IFN-γ dominates the T cells in preclinical (8–16 weeks) EAO testes, and the adoptive transfer of preclinical T cells causes preclinical EAO. In contrast, IL-17 dominates the T cells in clinical (>17 weeks) EAO testes, and the adoptive transfer causes clinical EAO. (2) Only the effector T cells from clinical EAO testes resist suppression by Tregs. (3) The transformation from Tregs into Th1 or Th17 cells occurs in vivo in clinical EAO testes. (4) In preclinical EAO, Effector T cells were located outside the blood–testis barrier and were noninvasive, while in clinical EAO testes, cells became invasive and penetrated BTB, thus depleting GCs [18]. Moreover, Terayama et al. observed a significant increase in the intratesticular mRNAs of IFN-γ and TNF-α in EAO mice [49]. In contrast, our studies show an increased activity and number of Th17 cells, as well as their cytokines (IL-17A, IL-21, IL-22) in human testes with ATCI, and a concurrent decreased level of Foxp3^+^ and IFN-γ^+^ cells was observed [13]. A possible explanation for this is that cytokines produced by Th17 cells in the testes can negatively regulate the function of Th1 cells. In addition, dendritic cells (DCs) might also be involved in orchestrating T cell immune responses in inflamed rat testes, as IL-12 or IL-23 secreted by DC could promote the development of Th1 and Th17 subsets in the local environment [48,50]. Based on our data and the other aforementioned studies, it is reasonable to propose that Th1 cells are augmented at the onset of orchitis, inducing anti-infection responses. However, in the chronic phase, Th17 cells dominate the Th cell subsets and maintain the inflammation state in the testes by inhibiting Tregs and crowding out other effector T cells. Collectively, Th cells in orchitis are involved in the impairment of the structure and spermatogenesis of the testes [51].

### 2.3. Cytotoxic T (Tc) Cells

Cytotoxic T (Tc) lymphocytes (commonly known as CD8^+^ T cells, CTL) constitute another essential part of the adaptive immune system, which includes antigens presented by MHC I, expressed by nearly all nucleated cells. After recognition and costimulation by other inflammatory cytokines, activated CD8 cells undergo clonal expansion and differentiation into effector CD8^+^ T cells, producing cytotoxic molecules, antiviral cytokines, and tumor necrosis factors [52]. The role of CD8^+^ T cells is to monitor the cells of the body, destroying any cell that is considered to be a threat to the body. Their targeting cell can be divided mainly into three classes: cells infected by viruses (or other pathogens), damaged or dysfunctional cells, and malignant tumor cells. After the clearance of target cells, the majority of effector CTLs will undergo apoptosis, and meanwhile, a small amount of them will turn into memory CTLs, preparing for the next war against the same enemy. Some CD8^+^ T cells have a similar cytokine pattern as CD4^+^ T cells, enabling a corresponding taxonomy, such as Tc1, Tc2, and Tc17. Human Tc17 cells were detected in a minor population of CD8^+^ T cells and were predominantly found in CD27^−/+^CD28^+^CD45RA^−^ memory subsets [53]. The three subsets of Tc cells, together with the three subsets of Th cells, can be allocated into three major kinds of adaptive immunity: (1) the activation of mononuclear phagocytes against intracellular microbes; (2) the activation of the mast cell, basophil, and eosinophil, as well as the induction of IgE antibody to combat against helminths and venoms; (3) the activation of mononuclear phagocytes, induction of epithelial antimicrobial response, and recruitment of neutrophils to protect against extracellular bacteria and fungi [54]. In recent years, microRNA has aroused an increasing amount of attention in immune research. Some microRNA act as modulators between T cells and tumor cells. For example, microRNA-155 are required for the response of CD8^+^ cells to virus and cancer [55]. These various immune responses aid in surveillance and maintain the homeostasis inside the testes, especially preventing tumorigenesis.

Tc cells have been identified in the testicular interstitium of rams [56], mice [20], rats [21], and humans [15]. Early studies demonstrated that the majority of T lymphocytes inside the testes are CD8^+^ T cells, the percentage of which is 4-fold greater than the percentage of CD4^+^ cells, whereas the percentage of CD4^+^ T cells are 2-fold higher in blood [11,20,57,58]. CD8^+^ T cell testicular subsets were demonstrated to be functionally associated with the number of testicular resident macrophages or Leydig cells and might be involved in the survival of grafts [59]. Pancreatic grafts in the testes, as an immune privilege site, show a significantly lower rate of rejection, compared with that in the kidney capsule. Interestingly, delayed intratesticular graft rejection is not due to a reduced proliferation of memory CD8^+^ T cells in that site, but rather due to increased apoptosis, mediated by the upregulation of Fas and CD30 on the cell surface [60]. Similarly, islet transplantation in the testes generates fewer memory CD8^+^ T cells but induces more Ag-specific CD4^+^CD25^+^ Treg cells than other tissues, such as the kidney capsule [61]. A possible explanation is that SCs, after transplant, activate testicular Treg cells through costimulatory molecule programmed death ligand-1 (PDL1), which binds PD1, expressed by Treg cells, and inhibits the proliferation and activation of CD8^+^ T cells [62]. Another likely explanation is that the majority CD8^+^ in the testes are monitoring and regulatory subsets, instead of cytotoxic CD8^+^ subsets.

On the other hand, the immune privilege may also provide an unexpected refuge for pathogens and tumor cells, which require CD8^+^ T cells for surveillance. Recently, an investigation in patients with testicular cancer suggests the development of cancer/testis-antigen (CTAg)-specific CD8^+^ cells in the blood, and orchidectomy reduces 89% T cells specific to melanoma-associated antigen (MAGE), one kind of CTAg. The presence of MEGA-specific CD8^+^ cells is proposed to be a positive indication for excellent clinical outcomes of patients with testicular cancer. Moreover, other research in testicular GC tumors also suggests a correlation between CD8^+^ T cells and recurrence-free survival [63]. In view of the pleiotropic role of microRNA in tumorigenesis and angiogenesis, CD8^+^ T cells may also have an antitumor role through the microRNA in the testes, such as microRNA-146a, which have an effect on both CD8+ responsiveness and tumor angiogenesis [64,65,66,67]. Studies in pig-tailed macaque testes with SIV infection demonstrated that although the number of testicular effector memory CD8^+^ T cells increased and SIV-specific CD8^+^ T cells were detectable 11 weeks after infection, their cytokine response to mitogen activation had been suppressed [68]. This result, on the other hand, suggests that the local immunosuppressive niche in the testes can restrict the ability of CD8^+^ T cells to respond to the virus.

Nonetheless, the sophisticated characterization of the phenotype and function of Tc cell subsets, such as Tc 1, 2, and 17 cells in the testes remains to be elucidated. As mentioned before, Jacobo et al. detected CD8^+^Foxp3^+^ Treg cells within the rat testes in the chronic phase of EAO, as well as IFN-γ- and TNF-α-producing CD8^+^ T cells [12,19]. The mechanism of why and how these CD8^+^ subsets play a role in chronic autoimmune disease remains to be further elaborated.

### 2.4. γδ. T Cells

Most mature T lymphocytes express T cell receptor (TCR) as the αβ chain, and other TCR-associated molecules, such as CD4 or CD8, a minor group of T cells (1–4% of T cells in adult human blood), express TCR as the γδ chain, a subunit mostly expressed in CD4^−^CD8^−^ T cells. In contrast to the low proportion of γδ T cells in the blood, the secondary lymphoid organs in epithelial tissues account for much higher proportions of γδ T cells, for example, about 10–20% in the reproductive tract of female mice [69]. Studies demonstrate that γδ T cells share the features of innate and adaptive immunity and might play a critical role in bridging two responses, providing antimicrobial and antitumor immunosurveillance [70]. Interestingly, γδ T cells respond earlier than αβT cells during infection and emerge again when pathogen loads start to drop, indicating their involvement in establishment and regulation during inflammatory response [71]. γδ T cells also have a similar taxonomy to αβ T cells, suggesting that they play a role in inflammation and tolerance as well. γδ T cells propagate the effects of IL-23 and IL-1β through IL-17 and IL-21 production in order to promote the development and expansion of Th17 cells [72]. Therefore, γδ T cells might act as a signal-amplifier to induce an adaptive immune response. On the other hand, Hahn et al. observed that while the Vγ1^+^ γδ T cell subset enhanced airway hyper-responsiveness (AHR), a disease correlated with autoimmune response, by increasing IL-5 and IL-13 and promoting eosinophilic infiltration after repeated exposure to ovalbumin (OVA), the Vγ4^+^ γδ T cell subset suppressed AHR [73]. These studies clearly reveal the heterogeneity inside the γδ T cell family and suggest that distinct γδ T cells can have an inflammatory or anti-inflammatory nature that modulates the pathogenesis of the disease.

γδ T cells have been found in murine testes [22] for a long time. γδ T cells residing in tissue interact with other innate and adaptive immune cells and modulate their function. Some display immunosuppressive activity at the inflammation site. An early mouse orchitis model showed that the depletion of testicular γδ T cells significantly increased the inflammatory response in both the infected and the contralateral testes [22]. Subsequent studies examined the repertoire of the infiltrating T cells in the EAO mice induced by *Listeria monocytogenes* and found an abundant presence of IL 17-producing Vγ6 γδ T cells, which form a fetal-derived γδ17 cell subset, playing an antibacterial role and reducing tissue damage under the inflammatory conditions [74]. Moreover, testicular γδ T cells presented in infection-induced and autoimmunity-induced inflammation display the same characteristics of immunoregulatory features [30]. Taken together, these data imply that testicular γδ T cells appear to be capable of reducing autoimmune response and play a surveillance role in antibacterial and auto-aggressive immune responses in mouse testes. Interestingly, γδ T cell responses are independent of the presence of bacteria or bacterial products [75]. These results indicate that the stimulus triggering the response of γδ T cells in the testes appears to be self-Ags or signaling induced during inflammation, instead of foreign infections.

While γδ T cells have been identified in human semen samples [76], few data are available concerning the presence and functional role of γδ T cells in human testes, compared to the well-studied counterpart in the female tract. Our examination of the control group and ATCI group of human testes by paraffin immunohistochemical analysis has not detected γδ TCR using a specific antibody (mouse monoclonal, clone γ3.20, Endogen) [13]. This may be due to the unreliable detection using formalin-fixed paraffin-embedded sections, and further examinations therefore need to be conducted in order to depict the frequency, characteristics, and functions of γδ T cells in human testes.

### 2.5. Natural Killer (NK) Cells and NK T Cells

While natural killer (NK) cells are classified under the innate immune lineage, they deserve to be mentioned here due to their regulatory roles and close relationship with NK T cells. NK cells are important effector lymphocytes that can (1) directly kill tumor and virus-infected cells, even in the absence of antibodies and MHC; (2) produce cytokines and chemokines, such as IFN-γ; (3) interact with other immune or nonimmune cells [77]. NK cells are tightly regulated by sensing the signal in the neighborhood, with the expression of a panel of inhibitory and activating receptors. MicroRNAs also modulate NK cells, delivering a message between NK and tumor cells, partly by microvesicle transfer [67]. NK cells can be roughly subdivided into two populations, based on the expression level of the surface markers, CD16 (FcγRIII) and CD56, in humans and that of NK1.1 or NK1.2 in C57BL/6 mice, with mutually exclusive cytokine-producing activities or cytotoxic abilities. However, in recent years, it has been well recognized that NK cells are more diverse, and some diversities are genetically determined by, for example, the KIR or environmental factors [78,79].

NKT cells are narrowly defined as a T cell lineage expressing NK lineage receptors. They display part of the immunoregulatory properties, as they enhance the immune response to tumors and infectious diseases, while inhibiting the cell-mediated immune reaction associated with autoimmune diseases and allograft rejection [80]. In contrast to NK cells, NKT cells are CD1d-restricted and express T cell receptors (TCRs)/pan-T marker CD3/surface immunoglobulin (Ig) B cell receptors. NKT cells can activate NK cells through IFN-γ secretion, and their cytotoxic ability is mainly restricted to the CD95/CD178 pathway, whereas NK cells rely more on perforin and granzyme-mediated mechanisms [81]. Absence or deficiency of NK T cells can lead to an increased autoreactive B cell activity and aggravated autoimmune disease progression. In addition, an increased NK T cell population has been demonstrated to modulate autoreactivity, suggesting that NK T cells play a significant protective role against autoimmunity [82].

NK and NKT cells have been found in normal rat, mouse, and macaque testes [23,57,83], although no researchers have found any NK cells in normal human testes [15]. However, this may be due to neglect upon inspection due to the heterogeneity of NK cells. The most widely used commercial antibody for detecting NK cells in human targets CD16, an antigen not expressed on all NK cells [84]. About 95% of CD8a^+^ NK cells regarded as having a mature status and possessing a high cytotoxic potential in the blood are CD56^dim^CD16^bright^. In contrast, a subpopulation of CD8^−^ NK cells and the majority of NK cells in the testes of macaques are CD16 negative, which is proposed to partly account for the lowered proneness to activation [83,85]. Moreover, in macaque and rat testes, the NK and NKT cell population is three- to five-fold higher than its counterparts in the peripheral. Together with a predominance of CD8^+^ T cells and a relatively minor proportion of CD4^+^ T cells in the testes, this indicates an enhanced immune surveillance and suppressed antigen-specific response in the testes [12,57,83].

The increased immune surveillance by the NK and NK T population might explain the lower incidence and mortality of GC tumors and seminomas in comparison with other cancers [86]. NK and NKT cells mediate antitumor responses and enable surveillance in various cancers, such as breast cancer and pancreatic cancer [87]. Studies have demonstrated the reverse correlation between the high amounts of NK cells and the incidence of metastases in patients with gastric, renal, and prostate carcinomas [88]. The highly infiltrated NK cells in the interstitial tissue of the testes might prevent the settlement of circulating tumor cells (CTCs) and decrease the further possibility of testicular cancer. It is possible that these NK cells also trap tumor cells inside the BTB by hindering the EMT of potential CTCs, thus facilitating effective orchiectomy and chemotherapy for testicular cancer.

To the best of our knowledge, NK cells have not been reported in the seminiferous duct under physiological conditions. Under pathological conditions, a shift of the CD4^+^ T cell and NK cell subpopulations in the testes and a significant increase in the CD56^+^ NK cells in the semen have been observed [89,90]. Like other immune privilege sites, an upregulated activating receptor and ligand for NK and downregulated inhibitory signals for NK cells under inflammation or infection might regulate the immune response in the testes [91,92]. The role of NK T cells in testicular immunity and therapeutic properties and the nature of testicular NK T cells will be promising areas of research in the future.

## 3. T Lymphocytes and Leydig Cells

Leydig cells are a flock of cells present in the interstitial region between seminiferous tubules. They are the primary source of androgens, which can bind the androgen receptors (AR) on peritubular myoid cells and SCs, thereby participating in spermatogenesis [93]. Leydig cells can bind and retain both normal and malignant T lymphoblasts on their surface, and the coculture of Leydig cells and T cells show the suppressive effect of Leydig cells on the proliferation of the latter [94]. Further data show that vascular cell adhesion molecule 1 (VCAM-1, also known as CD106), expressed by Leydig cells, may act as an adhesion-promoting molecule or a costimulatory factor for T cells, migrating to mouse testes through the interaction between CD106 and CD49d [95]. CD106 is also found to be expressed in Leydig cells and the basal parts of the SCs in human testes [96]. The above results indicate that human and mouse Leydig cells may recruit T cells from periphery blood by binding infiltrating T cells in the testes and may regulate T cell function through intimate contact.

Additionally, Leydig cells can also modulate T cell population and function though the hormone pathway, as ARs are widely expressed in lymphocytes. The destruction of Leydig cells by specific cytotoxin ethane dimethane sulphonate (EDS) can enhance the immune cell accumulation (CD8^+^ and CD4^+^) in inflammatory testes induced by dimethyl sulfoxide (DMSO) stimulation, while the replacement of testosterone can significantly inhibit the accumulation of CD8^+^ T cells in the acute stage. In the recovery stage, the addition of testosterone can decrease the population of both CD8^+^ and CD4^+^ T cells to lower than the control level potentially through cooperation with recovery Leydig cells. Moreover, the change of CD4^+^ T cells was found to closely follow macrophages in the testes, indicating the involvement of macrophages in Leydig–T cell interaction [97,98]. In subsequent studies, rat Leydig cells were further proved to be able to regulate CD8^+^ T cell traffic directly or indirectly via testicular macrophages. The number of testicular T cells, especially the number of CD8^+^ T cells, is positively correlated with the number of resident macrophages and is negatively correlated with subcutaneous testosterone implants in rat testes [59,99]. In addition, the depletion of testosterone though the disturbance of Leydig cells by EDS injection results in epididymal sperm granuloma and inflammatory immune infiltration (CD4^+^ and CD8^+^ T cells), which can be prevented through testosterone replacement [100]. Another experimental study on autoimmune encephalomyelitis elucidated the mechanism by which testosterone directly induces IL-10 production in CD4^+^ T cells during stimulation with anti-CD3 [101]. Apart from the inhibitory role of the activation of CD8^+^ and CD4^+^ T cells, evidence from the EAO rat model shows another way that androgen exerts an immunosuppression function indirectly by promoting the expansion of Tregs [102]. An in vitro culture shows that Splenic CD4^+^ T cells can be dose-dependently stimulated to express Foxp3 and secrete IL-10 in Leydig cell-conditioned media, while the addition of antiandrogen flutamide abolishes this effect [103]. The addition of androgen in cultured human Tregs shows that it can induce the Foxp3 expression by changing the acetylation status of histone H4 [104]. In turn, inflammation in the testes can destroy the Leydig cell group, and the elimination of CD8^+^ or γδ T cells diminishes the testicular inflammation, thus protecting the Leydig cells [105]. Recently, a significantly decreased number of Leydig cells in the testes, along with T lymphocyte infiltration, was observed under infection with COVID-19, and the expression of the virus receptor angiotensin-converting enzyme 2 (ACE2) was proved to be highest in the testes [106,107]. Another preprint study on COVID-19 by Ma et al. found a similar T lymphocyte infiltration, including CD3+ T lymphocytes and CD20^+^ B lymphocytes, and RNA-seq analysis reveals upregulated inflammation response-related pathways. Similarly, CD3^+^ T lymphocyte infiltration and IgG precipitation was observed in the testes of six patients, who died of severe acute respiratory syndrome (SARS), while the SARS viral genomic sequence was absent, implying the role of circulating T lymphocytes in secondary testis inflammation [108]. Gathering these results, we can systematically depict the role that Leydig cells play in the immunity of the testes and indicate that androgens secreted by Leydig cells can regulate the infiltration and activities of T cells through both direct and/or indirect modes within the testes and adjacent draining lymph nodes.

## 4. T Lymphocytes and Sertoli Cells

SCs are the essential constituent part of seminiferous tubules, providing structural and functional support for the development of maturing GCs. In addition, they act as immunological sentinels of spermatogenesis by not only forming the blood–testis barrier, but also expressing immunoregulatory factors [2]. Sertoli cell/islet cograft shows that the infusion of SCs into islet transplantation effectively prolonged the survival of islet grafts by reducing CD4^+^ and CD8^+^ T cells but increasing the CD4^+^ or CD8^+^ Tregs [109,110]. Interestingly, while syngeneic SCs help to impede CD8^+^ T cell infiltration through the formation of a tubule structure, allogeneic SCs fail to form this structure and prevent the infiltration of active immune cells, thus having a detrimental effect on islet graft survival [111]. This result suggests that the histocompatibility between SCs and surrounding immune cells might be indispensable for their recognition and communication. Meanwhile, it seems that transplanted SCs generated an anti-inflammatory cytokine milieu with an increased number of Treg and decreased IL-17-producing CD4^+^ T cells, thus changing the immune response from destructive to protective [112]. The protection of syngeneic islets transplanted to the contralateral kidney by SCs suggests that the immune regulatory role of SCs is exerted both locally and systemically.

How SCs function as imperative immune regulators in the testes is not clear yet. Here, we discuss some potential mechanisms of SCs in inducing immune tolerance, directly or indirectly. Early research on culturing spleen lymphocytes in Sertoli-conditioned media show that product(s) secreted by SCs can dose-dependently inhibit the proliferation of lymphocytes, and the synthesis of their product(s) are follicle-stimulating hormone (FSH)-dependent. The inhibition leads to IL-2 depletion; nevertheless, that exogenous IL-2 fails to reverse the inhibition indicates that Sertoli secretion inhibits both IL-2 production and the responsiveness of T lymphocytes [113]. A later study by Cesaris et al. further elucidates the inhibitory mechanism of Sertoli secretion, which arrests lymphocytes in the G1 phase of the cell cycle [114]. In this century, the truth about these secretions was gradually unveiled. Sipione et al. found that testicular SCs are capable of secreting granzyme B inhibitors, which form part of the destructive arsenal of CD8^+^ T cells [115]. Fallarino et al. found that SCs can produce indoleamine 2, 3-dioxygenase (IDO), TGF-β, and complementary inhibitors, and they are an important inducer of inflammation and the subsequent immune response, when they are used as isolated SCs to treat experimental type 1 diabetes [112]. The most recent data further prove the immune regulatory role of IDO in EAO rats [116]. Microarray analyses and qRT-PCR in SCs demonstrate that SCs express serine protease inhibitor (SERPIN)G1, which targets the initial step of the complementary cascade activation, thus preventing further amplification [117]. Moreover, galectin-1 is expressed in SCs and plays a proinflammatory role through MAPK signaling in the early or acute stage of chronic inflammation. It is also able to promote the differentiation of tolerogenic DCs and Tregs, suggesting the dual role of SCs in testicular immunity [118]. On the other hand, these SC-secreting cytokines, especially TGF-β, can regulate the pluripotency and proliferation of germline and SCs themselves and may therefore impact sperm formation under chronic inflammation stimulation [119].

Besides these immune regulatory molecules, SCs were also proposed to directly interact with some immune cells, although the evidence and details remain limited. Cocultures of mouse SCs and lymphocytes show that SCs strongly upregulate the negative costimulatory ligand B7-H1 to weaken the proliferation of CD8^+^ T cells in response to IFG-γ. Moreover, these SCs were also found to express MHC-II and function as nonprofessional antigen-presenting cells (APCs) that mediate the conversion of Tregs from CD4^+^CD25^−^ T cells to CD4^+^CD25^+^Foxp3^+^ T cells [120]. Recently, a novel mechanism was described, by which SCs induce functional CD4^+^CD25^+^Foxp3^+^ Tregs in vitro by JAGGED-1 via Notch pathway signaling [121]. Additionally, the lymphocyte endothelial epithelial-cell adhesion molecule (LEEP-CAM) is found to be expressed in normal SCs in human testes, which is a molecular mediating lymphocyte adhesion to some epithelial cells. The loss of LEEP-CAM is positively correlated with impaired spermatogenesis, including end-stage tubular damage, resulting from the infiltration of T cells [122].

The expression of Fas/FasL during testicular development and its distribution within the testes is still an ongoing debate. However, it is generally assumed that the expression of FasL by testicular SCs is implicated in the maintenance of testicular immune privilege and prolonged survival of allogeneic grafts by inducing the apoptosis of T cells and may be negatively regulated by TNF-α, a proinflammatory cytokine [109,123]. Another study focused on the adverse impact of fluoride on SCs, indicating that a decreased FasL lever in fluoride-treated SCs are negatively correlated with the viability of lymphocytes and are positively correlated with their apoptosis [124]. These data demonstrate that SCs can modify T cell responses by producing immune regulating factors and possibly through direct interaction with immune cells. Most importantly, the immunoregulatory properties of SCs appear to be an imperative and more important function than their contribution to the blood–testis barrier.

## 5. T Lymphocytes and Spermatogenesis

Spermatogenesis is a complex process, and many factors are involved in regulating this process, including innate or adaptive immune cells, cytokines, chemokines, and somatic cells. For example, IL-6 and IL-1 can regulate SCs and spermatogenic cell development, and both SCs and Leydig cells are able to produce IL-6 under the stimulation of IL1 in vitro [125]. The sophistic mechanism to maintain spermatogenesis and destroy the milieu to impair this process remains relatively unclear. Here, we focus on T lymphocytes and discuss their positive or negative impact on spermatogenesis under physiological conditions or inflammatory conditions, respectively.

In normal testes, the number of GCs is balanced by cell proliferation and apoptotic cell death, discarding defective GCs and removing excess GCs to fit the supportive capacity of SCs. The dynamic balance of the GC population is accurately controlled by intracellular signals, such as Bax and Bcl-xL, or extracellular signals from immune and somatic cells. IL-6, TNF-α, and the soluble form of FasL (sFasL), which are produced by CD4^+^ and CD8^+^ T cells, as well as other immune cells distributed in the testicular interstitium, are able to enter the seminiferous tubules and are involved in GC apoptosis in normal testes [126]. The dysfunction of these apoptotic signals in the testes fails to prevent excessive spermatogenesis, thus leading to an abnormal testes size and defective sperm status [127]. Moreover, BTB is incomplete in the rete region, making it a particular site for potential communication between GCs and T cells. Tregs in the testes are distributed in the testicular interstitium, mainly under the subalbuginea or in the peritubular areas. As mentioned above, Tregs can recognize the sperm antigens leaked from tubules and induce immune tolerance in the testes, thus protecting spermatogenesis.

**Figure 2:**
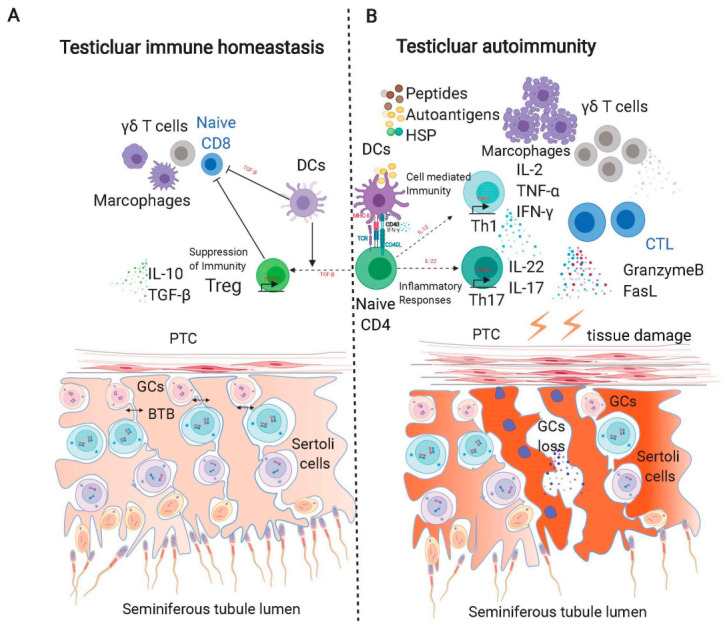
Hypothetical schematic model of testicular immune privilege under normal (**A**) and inflammatory (**B**) conditions. A: Under normal conditions, Th cells are tightly controlled by Tregs through multiple mechanisms. B: In the presence of chronic testicular inflammation, resulting from a temporary or silent infection, necrosis of testicular cells can lead to the release of self-antigens (e.g., HSP, ODF-2), peptides, and/or of pathogen-associated molecular patterns, i.e., LPS, which can enhance the maturation and population of antigen-presenting cells and Mø in the testes, thus further activating Th17 cells and triggering autoimmunity. CTL, cytotoxic T lymphocytes; DC, dendritic cell; GC, germ cell; HSP, heat shock protein; IDO, indoleamine 2,3-dioxygenase; T, testosterone; CCL, chemokine ligand; FasL, Fas ligand; IL, interleukin; IFN-γ, interferon; LC, Leydig cell; Mø, macrophage; ODF-2, outer dense fiber protein 2; PTC, peritubular cell; SC, Sertoli cell; TGF-β, transforming growth factor β; TNF-α, tumor necrosis factor α; Th, T helper cell; Treg cell, regulatory T cell.

Viruses and bacteria are regarded as common factors causing male infertility [128]. However, even after the elimination of bacteria in the mouse model or during the chronic inflammation after infection in humans, the seminiferous epithelium remains destroyed, and spermatogenesis remains disrupted in the inflammatory testes, suggesting chronic inflammation as the main cause for infertility [27]. Actually, in infertile patients with a history of orchitis, a statistically increased antisperm antibody had been detected in blood and semen, suggesting a profound impact of inflammation on male fertility [129]. Under an inflammatory status, spermatogenesis can be disturbed: GCs undergo apoptosis and sloughing due to excessive proinflammation cytokines, exorbitant apoptotic signals, and an atrophic seminiferous structure with peritubular fibrosis. Adhesion molecules on the endothelial cells, such as hyaluronan and CD106, are firstly upregulated to attract leukocytes, especially T lymphocytes, which express CD44 and CD49d, respectively, which help them to extravasate into the interstitial space. Chemokine ligands, like CCL2, CCL3, and CCL4, further arrest and accumulate these leukocytes inside the testes [126,130,131]. A recent study indicates that human testicular peritubular cells expressing Toll-like receptors (TLR) and proteoglycan biglycan (BGN) can secret IL-6, monocyte chemo-attractant protein-1 (MCP-1), and pentraxin 3, orchestrating immune events around seminiferous tubules [132]. The accumulation and activation of these lymphocytes enhanced the secretion of proinflammatory or proapoptotic cytokines, such as TNF-α, IL-6, and FasL. TNF-α is mainly produced by activated Th1, and it targets spermatocytes that express TNFR1. The number of TNFR1^+^ spermatocytes increased during orchitis, as well as their apoptosis, coincide with the aggravated apoptosis of GCs in vitro by TNF-α stimulation [133]. A high level of IL-6 and IL-17 inside the testes is capable of perturbing the tight junction barrier of SCs, thus promoting the release of GCs inside seminiferous tubules [134,135]. Intriguingly, a significant increase in IL-6R^+^ GCs co-occurred with aggregative testis damage and GC apoptosis, suggesting the proapoptotic role of IL-6, in addition to junction disturbance [136]. The enhanced spermatic antigen release by IL-6 disturbance initiates the activation of Tregs; however, activated Tregs fail to suppress inflammation due to dominated effector T cells [12]. Meanwhile, these antigens further activate immune cells in the interstitium and may induce FasL expression in rat CD4^+^, CD8^+^ T cells, or human monocytes [137]. The increased FasL can easily pass the impaired BTB and target Fas on the spermatocytes and spermatids, the expression of which is enhanced during chronic inflammation, resulting in increased GC apoptosis (Figure 2). An ex-vivo experiment, where GC Fas activation significantly induced apoptosis, proves the in-vivo efficiency of the FasL-Fas system [138].

The infiltration of T lymphocytes and the impairment of germinal epithelium and GC antigen release were also observed in biopsies of human testes and are frequently associated with male infertility [138]. Our results showed the presence of Th17 cells and their secretions of IL-17, IL-21, and IL-22 in the testes of azoospermic patients with chronic inflammation [13]. We also detected TNF-α in the ejaculates of patients with chronically reproductive tract inflammation and found that TNF-α can induce spermatozoa apoptosis and impair sperm motility [139]. Moreover, aberrant sperm DNA methylation patterns have been associated with male subfertility and oligospermia, and functional analysis reveals that a significant loss of methylation genes is involved in inflammation and immune response [140]. These data emphasize the important role of T lymphocytes in maintaining spermatogenesis in normal testes, whereas under inflammation or infection, it may damage spermatogenesis both by inducing apoptosis and interfering with epigenetic modification, which may cause male subfertility or be related to unhealthy offspring [141], respectively.

## 6. Therapeutic Options for Chronic Orchitis

Chronic orchitis, a major cause of male infertility, occurs asymptomatically in the majority of patients and is difficult to diagnose, owing to the absence of specific seminal or serological markers; therefore, chronic testicular inflammation is frequently ignored [24]. Further, because of the lack of well-defined diagnostic criteria, relevant guidelines for treatment are still limited [28]. A primary strategy of causal treatment for chronic testicular inflammation is early detection, which can prevent deleterious progress, causing male fertility disorder. In view of this, the determination of antitesticular antibodies could be a promising tool, as the characteristic antibodies are likely to be produced during chronic inflammation [142]. Recently, the advancement of new technology, such as full-spectrum cytometry and single-cell sequencing, allows us to analyze samples more comprehensively and precisely [143,144]. The multidimensional application of these techniques might give us unique characteristic information for chronic orchitis, like fingerprints, enabling precise and personal therapy strategies. Evaluations of the ratio between lymphocyte subsets and/or other immune cells have been attempted, as it may be a candidate indicator for latent male genital inflammation [145]. Further, the understanding of the relationship between inflammation and spermatogenesis may provide cues for therapies, such as (1) enhancing the function of Tregs and recovering the balance between Treg and Teff cells to restrict the function of Teff cells; (2) depleting effector T cells and/or neutralizing their cytokines (e.g., TNF-α IL-2 and IL-17) to decrease the inflammatory lesions and autoimmune response; (3) transferring tolerogenic APCs (macrophages and DCs) and anti-inflammatory factors (e.g., IL-23) to alleviate the polarization of naive T cells; (4) activating or inhibiting γδ T cells and NK T cells, respectively.

## 7. Conclusions

The publications reviewed in the present study show the subtle equilibrium between immune privilege and inflammation inside the testes. An imbalance in favor of one or the other would cause testis-related dysfunction or disease. T lymphocytes play an imperative role in testis immunity and orchestrate other immune cells and stromal cells inside the testes, thus maintaining a tolerance status in terms of the physiological condition and arousing an immune response under threat of infection and tumorigenesis (Table 1). However, an aberrant immune response may be sometimes induced in the testes due to recurrent infection, genetic deficiency, or physical damage, thus resulting in chronic inflammation or autoimmune orchitis. While in this review, we summarized the role of different T cell subsets in normal testes and their contribution to testicular inflammation and impaired spermatogenesis, the sophisticated relationship between these cells remains to be investigated. Treg cells mainly act as suppressive cells that lead to immunotolerance to sperm antigens, while they fail to correct excessive inflammation because of the overwhelming effector T cells. Th1 cells and Th17 cells dominate the onset and chronic phase of inflammation, respectively, secreting their specific cytokines. These cytokines carry out potential suppressive effects on the opposite subsets, suggesting unique immune profiles in different inflammation stages. CD8^+^ T cells occupy the majority of T cells in normal testes, and their cooperation with NK (T) cells consolidate immunosurveillance in the interstitial space. However, excessively activated cytotoxic CD8^+^ T cells are prone to inducing autoimmunity, thus requiring prudent regulation by other immune cells or a subtype of CD8^+^FoxP3^+^ regulatory cells. Leydig cells and SCs perform locally as powerful regulators to harmonize immune response and spermatogenesis. Leydig cells can both recruit T cells and suppress T cell activation, with the selection depending on homeostasis or inflammation. SCs not only build a barrier, but also communicate with T cells and other immune cells to maintain a tolerant milieu for GCs, either indirectly by the molecules, granzyme B, IDO, and TGF-β, or directly by B7-H and the Fas/FasL system. The coordination of these cells guarantees regular spermatogenesis, while disorder among them induces a destructive testis structure and infertility. Nevertheless, further studies are required to investigate the onset signal, the phenotypic and functional changes of Treg cells, and the roles of the cytokine profile driving the shift from homeostasis to inflammation. Moreover, γδ T cells and NK T cells, as well as other potential immune cells and cytokines, remain to be characterized in human testes.

Besides, as most T cells share a similar function and distribution in human and animal models, animal chronic orchitis models, such as EAO, would provide some references for us to investigate the mechanism of chronic inflammation in human testes. However, interspecific differences deserve our attention, because the phenotype characterized by immune markers, such as Foxp3^+^ T cells, can represent a divided immune function in humans and other species, which has been discussed in this review.

Finally, considering that the environmental impact on the father can be transmitted to offspring through epigenetic alteration on spermatozoa, immune regulation and immune privilege might play an important role in this process as well, and they have gained more attention in recent years. A close inspection of testicular inflammation and spermatogenesis may offer a deep insight into the mechanism of male infertility and help us to understand how to acquire healthy sperm, both in vivo and in vitro.

## Figures and Tables

**Table 1:** T lymphocytes in normal human, mouse, and rat testes.

T Cell Subsets	Human	Mouse	Rat	References
Treg cells	+ +	+ +	+ +	[13] H
				[14] M
				[12] R
Th1 cells	+	+	+	[15] H
				[16] M
				[17] R
Th17 cells	+	+	+	[13] H
				[18] M
				[19] R
Tc cells	+ +	+ +	+ +	[15] H
				[20] M
				[21] R
γδ T cells	ND	+	?	[13] H
				[22] M
NK T cells	?	(+)	+	[23] M,R

Treg cells: regulatory T cells; Th cells: helper T cells; Tc cells: cytotoxic T cells; NK T cells: natural killer T cells; + +: strong positive; +: positive; (+): weak positive; ?: data unavailable; ND: not detected; ^H^: reference for human testes; ^M^: reference for mouse testes; ^R^: reference for rat testes.

## Abbreviations

ACE2	Angiotensin-converting enzyme 2
Ag	Antigen
AHR	Airway hyper-responsiveness
AIRE	Autoimmune regulator gene
APCs	Antigen-presenting cells
AR	Androgen receptors
ATCI	Azoospermic testis with chronic inflammation
BGN	Proteoglycan biglycan
BTB	Blood-testis barrier
CTAg	Cancer/testis-antigen
CTCs	Circulating tumor cells
DCs	Dendritic cells
DMSO	Dimethyl sulfoxide
EAE	Experimental autoimmune encephalomyelitis
EAO	Experimental autoimmune orchitis
EDS	Ethane dimethane sulphonate
GC	Germ cell
IDO	Indoleamine 2, 3-dioxygenase
IFN-γ	Interferon-γ
Ig	Immunoglobulin
IL	Interleukin
ILN	Lymph nodes from the immunization site
LEEP-CAM	Lymphocyte endothelial epithelial-cell adhesion molecule
MAGE	Melanoma-associated antigen
MAPK	Mitogen-activated protein kinase
MGCA	Meiotic germ cell antigens
MHC	Major histocompatibility complex
NK	Natural killer
OVA	Ovalbumin
PD1	Programmed cell death protein 1
PDL1	Programmed death ligand-1
SC	Sertoli cell
SEPRIN	Serine protease inhibitor
sFasL	Soluble form of FasL
Tc	Cytotoxic T
TCR	T cell receptor
Tfh	T follicular helper
TGF-β	Transforming growth factor-β
Th	T helper
TLN	Lymph nodes of testis
TLR	Toll-like receptors
TNF-a	Tumor necrosis factor-a
Treg	Regulatory T
VCAM-1	Vascular cell adhesion molecule 1
